# Soil quality under different land uses in eastern India: Evaluation by using soil indicators and quality index

**DOI:** 10.1371/journal.pone.0275062

**Published:** 2022-09-22

**Authors:** Parijat De, Shovik Deb, Dibyendu Deb, Somsubhra Chakraborty, Priyabrata Santra, Puspendu Dutta, Anarul Hoque, Ashok Choudhury

**Affiliations:** 1 Uttar Banga Krishi Viswavidyalaya, Cooch Behar, India; 2 ICAR-Indian Agricultural Research Institute, Gogamukh, Assam, India; 3 Indian Institute of Technology Kharagpur, Kharagpur, India; 4 ICAR-Central Arid Zone Research Institute, Jodhpur, Rajasthan, India; ICAR National Rice Research Institute, INDIA

## Abstract

Indian soils are inherently poor in quality due to the warm climate and erosion. Conversion of land uses like forests to croplands and faulty management practices in croplands further cause soil degradation. This study aimed to understand the extent of these impacts in a small representative part of eastern India, covering Himalayan terai and nearing alluvial plains. Soils were collected from (i) forests, (ii) croplands (under agricultural practices for more than 50–60 years) and (iii) converted lands (converted from forests to croplands or tea gardens over the past 15–20 years). Different soil quality indicators were assessed and soil quality index (SQI) was generated to integrate, scale and allot a single value per soil. Results indicated that continuous organic matter deposition and no disturbances consequence the highest presence of soil carbon pools, greater aggregation and maximum microbial dynamics in forest soils whereas high application of straight fertilizers caused the highest available nitrogen and phosphorus in cropland soils. The SQI scorebook indicated the best soil quality under forests ($\bar{x}$ 0.532), followed by soils of converted land ($\bar{x}$ 0.432) and cropland ($\bar{x}$ 0.301). Comparison of the SQI spatial distribution with land use and land cover confirmed the outcome. Possibly practices like excessive tillage, high cropping intensity, no legume in crop rotations, cultivation of heavy feeder crops caused degraded soil quality in croplands. This study presented an example of soil quality degradation in India due to land use change and faulty management practices. Such soil degradation on a larger scale may affect future food security.

## 1. Introduction

Soil quality can be defined as soils’ potential to optimally function within the ecology and land-use boundaries towards biological productivity and proper ecosystem services [1, 2]. The concept of soil quality appeared very prominently in the 1990s and following that emerged the need for tangible soil indicators to measure it [3]. Scientists considered a wide range of measurable physical, chemical and biological soil parameters as the quantitative indicators of soil quality like soil organic carbon (C), C fractions, texture, electrical conductivity (EC), nutrient contents, pH, aggregate stability, soil microbial characterization etc. [4–8]. The assessment of soil quality by understanding the threshold range of these indicators in a certain ecology also shows the sustainability potential of that soil [9].

Changes in land use and land cover (LULC) causes slow but permanent alterations in the individual soil parameter and in overall soil quality [10]. Under continuous anthropogenic pressure, shifts in LULC like agricultural expansion, urbanization have become a recent global trend. Widespread deforestation has been observed in tropical areas in the last few decades [11]. And these changes in land use, land management and landscape dynamics influenced soil quality very prominently during this short time [4]. Studies showed that conversion of forest area to cultivated lands under anthropogenic pressure results in severe degradation of soil quality in terms of porosity, aggregate stability, soil C and nitrogen (N) content, humus quality etc. [12–14]. While, conversion of forests into grasslands and tea gardens caused a noteworthy decline in soil C content and overall soil quality [14, 15], alteration of grasslands to croplands led to the further curb in organic C stock as well as soil microbial biomass C, root biomass and root C [16]. Although a lot of studies established a thumb rule of degradation in soil quality by converting natural land to croplands, the impact of artificial fertilizer application in the agricultural system sometimes improves certain soil indicators [17]. Metadata analysis indicates that even within croplands, soil quality indicators change with crop types (annual or perennial) and management practices [18, 19]. To accurately comprehend the quality of soils, there is a need to establish a quality index, which integrates different soil parameters along with their stratification and proper allotment [20, 21]. A numerical soil quality index (SQI) that can provide a single value for each soil by combining the impact of all quantitative parameters will be considered and termed as indicators now onward [7, 22]. This SQI can also evaluate and reflect the soil degradation due to land-use changes and management practices [21].

This study used SQI to understand the soil quality of an area of eastern India. It was important as this area is unique in terms of LULC heterogeneity. Several forests of national importance are situated here and it is also a famous tea-belt. In the last few decades, the increase in population pressure resulted in many-fold increases in croplands and urban settlements in this region. New tea gardens were also established in the forest fringe areas [23]. As a result, the virgin lands witnessed several folds of anthropogenic encroachments and interventions [24]. We hypothesized that LULC change of this area significantly affected the soil quality, which can be accurately measured by SQI. The objective of this study was to use SQI to understand the impact of land use on soil quality. For proper visual comprehension, spatial distribution of soil quality was also studied.

## 2. Materials and methods

### 2.1. Study area and soil sampling

**Fig 1:**
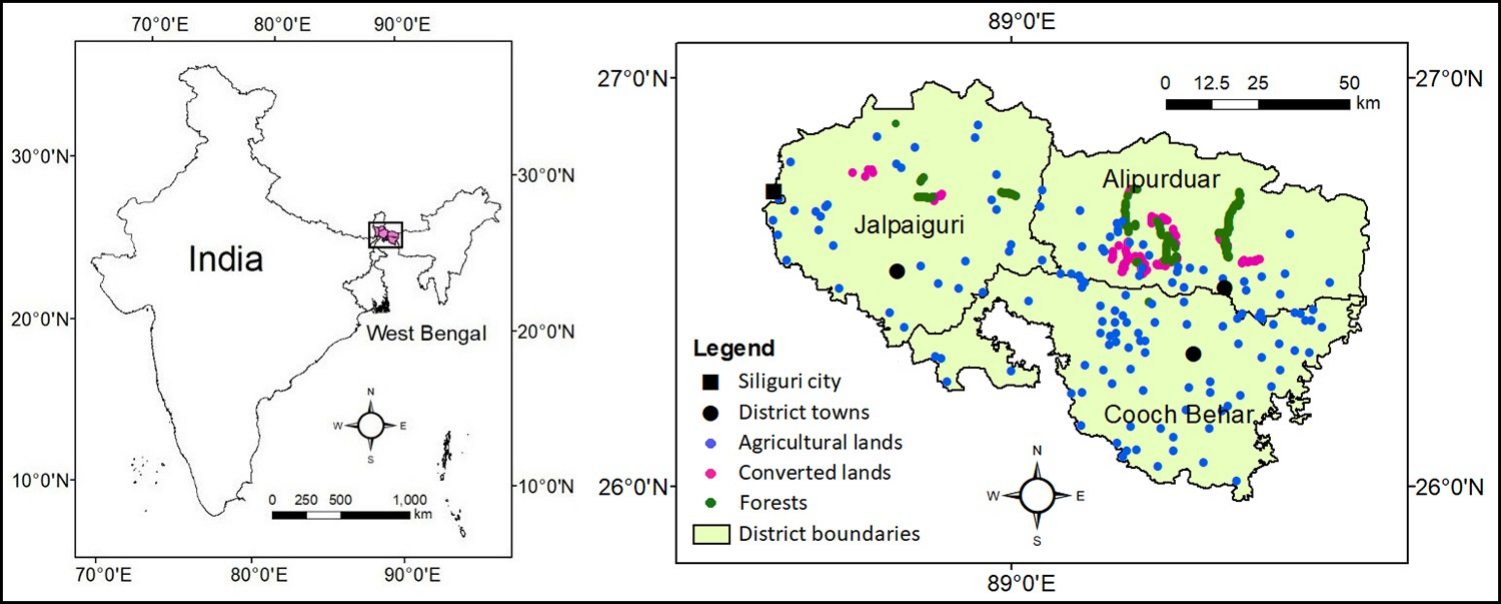
Study area indicating soil sampling locations.

This study was conducted in the northern part of West Bengal state (26–27°N; 88–90°E) of India, south to the Bhutan Himalaya and north to the plains of Bangladesh (Fig 1). It comes under humid to per-humid bioclimate [25] and covers three entire districts *viz*. Cooch Behar, Jalpaiguri and Alipurduar. Physiographically the area can be divided into Himalayan Terai and flat plains, formed by the alluvial deposition Teesta, Torsa, Jaldhaka, Sankosh, Mahananda rivers. These all are tributaries of the Brahmaputra River and being in their upper and middle courses, deposit mainly sand and silt (coarse particles) in this region. The soils of the area are Entisols (in floodplains) and Inceptisols (above the floodplains) [26]. There are several mixed semi-evergreen forests and grasslands here, including national parks (like Buxa Tiger Reserve, Jaldapara, Garumara, Neora Valley) and wildlife sanctuaries (such as Chapramari, Jorepokhri, Mahananda). Croplands, tea gardens and urban sprawls are the other types of land uses. In Terai and alluvial plains, the cropping system is solely rice-based and the average cropping intensity is >200%. An increase in population pressure, the subsequent need for the more cultivated area and socio-economic development has caused a change in LULC of this region in the last 2–3 decades with the rapid conversion of open, unprotected forests to newly cultivated lands (dominantly) and tea-gardens (in some places) [23, 24]. These converted lands are situated at the fringe of present-day forests and cultivation practices have started only in the last 15–20 years. However, the cropping system of converted lands is a bit irregular.

A total of 450 surface soil samples (0–20 cm depth) were collected from the area using hand trowel [27]. Each of these soils was a composite sample, prepared from 4 soil subsamples of the same/nearby fields. Soils were collected from 3 different land-use ecologies *viz*. (i) forests, (ii) croplands (under agricultural practices for more than 50–60 years) and (iii) converted lands (converted from forest to croplands or tea gardens over the past 15–20 years) with 150 samples from each of the ecology. Deliberately, the sampling was restricted to the Terai and alluvial plains only (Fig 1), as the Himalayan hills have very different physiography and subsequent soil-forming process. In some places (mainly under forest), the thin surface organic layer (O horizon) was gently scraped aside to collect the mineral soil only. The soil samples were collected during the pre-monsoon season (April-May 2016) when the fields were fallow and the soils were dry.

### 2.2. Land use and land cover analysis from satellite data

Cloud free Landsat 8 OLI data (collection 2 level 2) of 10th March 2016 (Path 138, Row 41, 42) and 17th March 2016 (Path 139, Row 41, 42) were downloaded from the United States Geological Survey data hub (https://earthexplorer.usgs.gov). No preprocessing was done as these data were already geometrically, radiometrically and atmospherically corrected. After mosaic formation and selection of area of interest, supervised classification was conducted on the image using the maximum likelihood algorithm in ERDAS Imagine 2016 software [24, 28]. A detailed ground survey was done for the selection of training sites. Different prominent LULC classes were identified and to avoid misclassification, they were cross-checked with ground observations and the author’s prior knowledge about the study area. Finally, spatial filters (3 × 3) were applied to eliminate the isolated pixels [24].

### 2.3. Soil analysis

The collected soil samples were air-dried. After removing the visible plant roots, clods were broken and the soils were passed through 5.0 mm and 2.0 mm sieves. For each soil, a subsample of <2.0 mm size was used for Physico-chemical characterization [29]. Soil pH was measured by a digital pH meter (Systronics, Model 4381). Soil cation exchange capacity (CEC) was estimated using 1.0 *N* ammonium acetate (NH4OAc) at pHw 7.0 [29], while soil exchangeable Al was estimated by KCl extraction and titration [30]. Soil texture was determined using the international pipette method [31]. Total C of bulk soil was estimated using Vario EL III elemental analyzer (Elementar, Germany). Inorganic C was measured via pressure calcimeter [32] and the difference between was total C and inorganic C was considered as soil organic C. Soil available N and available phosphorus (P) were estimated using Kjeldahl apparatus and spectrophotometer (Shimadzu UV-Vis 1800), respectively [33, 34].

Soil samples were physically fractionated into aggregates and as per their densities. For density fractionation, sodium polytungstate (SPT) was used as the heavy liquid [35]. After adjusting the density to 1.6 g cm^-3^, the samples were shaken in repeated cycles and filtered by Whatman glass filter paper (0.7 *μ*m pore size) to separate light and heavy density fractions [36]. The fractions were further analyzed in Vario EL III elemental analyzer (Elementar, Germany) to measure C associated with heavy and light fractions. The soil subsamples of 2.0 to 5.0 mm diameter (separated during air-drying) were used for aggregate analysis by dry sieving [37]. For each sample, 100 g soil was mounted on a nested set of sieves in a dry sieve shaker (of 2.0, 1.0, 0.5, 0.25, 0.1, 0.05 mm diameters). The sieves were mechanically shaken vertically for 2 mins with 10 mm amplitude. The weight of the soil samples, collected in each of the sieves, were measured and then used to calculate aggregate stability indices like mean weight diameter (MWD) and geometric mean diameter (GMD) as per the following equations [36, 38].


$$MWD=\sum_{i=1}^{n}{\bar{X}}_{i}W_{i}$$ (1)

$$GMD=exp\left[\sum_{i=1}^{n}log\;{\bar{X}}_{i}\;W_{i}/m\right]$$ (2)

Where, ${\bar{X}}_{i}$ and Wi represented the mean diameter (mm) of each size fraction and proportion of the total sample weight to corresponding size fraction, respectively. The m and n represented mass of sample and number of size fractions, respectively. These samples, collected in the sieves, were then oven dried at 65°C and analyzed in Vario EL III elemental analyzer (Elementar, Germany) for aggregate associated C.

Soil microbial activity was measured by enzymatic activities, as represented by fluorescein diacetate hydrolysis (FDA-HR) assay [39]. Air-dried soils were mixed with potassium phosphate buffer and FDA [40]. The mixture was incubated, filtered through a cellulose nitrate membrane filter (0.45 *μ*m pore size) and the extract was measured in 492 nm using a spectrophotometer (Shimadzu UV-Vis 1800). The reading was converted to μg g^-1^ h^-1^. In contrast, separate subsamples of all the soils were stored at 4°C in the field-moist condition. These soils were used to determine soil microbial biomass C following the chloroform fumigation-extraction method [41]. Soil microbial quotient was computed as the percentage of organic C present as microbial biomass C [29].

### 2.4. Development of soil quality index

For the development of SQI, the numbers of soil indicators were reduced following their high eigenvalues (≥1) in principal component analysis (PCA). The variables with the highest eigenvectors and absolute factor loadings (corresponding to each of the principal components (PC)) were only considered to develop a minimum data set (MDS) [21, 42]. The multivariate correlation coefficient was then calculated to see which variables could be considered redundant and could therefore be eliminated [42]. In case of variables with high correlation (Pearson’s correlation coefficient >0.6), only the variable with higher eigenvector loading was retained for the MDS [42, 43]. Next, these selected variables were transformed to indicator scores (within a range of 0–1) as per the following equation:

$$y_{i}=\frac{x_{i}-min\;(x)}{\max\;(x)-min\;(x)}$$ (3)

Where, *x*_*i*_ is the original variable and *y*_*i*_ is the transformed indicator score [7]. Next, they were used to build an SQI through weighted additive function [7, 44]. Here, the weight of any variable was calculated as the amount of variation (%) in the total data set explained by that PC, divided by the cumulative variation % explained by all the PCs with eigenvalues ≥1. It resulted in the generation of coefficients for that variable [42]. Following the addition of weight to the soil quality indicators, SQI was built as follows:

$$SQI=\sum_{i=1}^{n}Si\times Wi$$ (4)

Where, S_i_ and W_i_ are the selected soil quality indicators and their corresponding weight, respectively and n is the number of indicators.

### 2.5. Mapping of soil quality index

The spatial variability of the SQI was estimated through geostatistical techniques in ArcGIS 10.8.1 software (ESRI Inc., USA). In semi-variogram (*γ*(*h*)), the average dissimilarity between the data separated by a vector (*h*) was estimated using following equation [45]:

$$\gamma (h)=\frac{1}{2N(h)}\sum_{i=1}^{N(h)}[z(x_{i})-z(x_{i}+h){]}^{2}$$ (5)

Where, *z*(*x*_*i*_) is the value of the variable *z* at location of *x*_*i*_, *h* is the lag distance or separation distance and *N*(*h*) is the number of pairs of sample points separated by *h*.

Next, the semi-variogram were fitted in standard models using weighted least square technique and three spatial variations parameters were calculated *viz*. nugget (*C*_0_), sill (*C*+*C*_0_) and range (*a*). Among the four standard semi-variogram models (Linear, Exponential, Spherical and Gaussian), the Gaussian was found as best fit model as per minimum residual sum of square (calculated in gstat auxiliary packages in R software) [45, 46]. Mathematical expressions of the Gaussian semi-variogram models is given below:

Gaussian model:

$$\gamma (h)=C_{o}+C\;\left[1-exp\;\{\frac{{-h}^{2}}{a^{2}}\}\right]\;for\;h\geq 0$$ (6)

**Fig 2:**
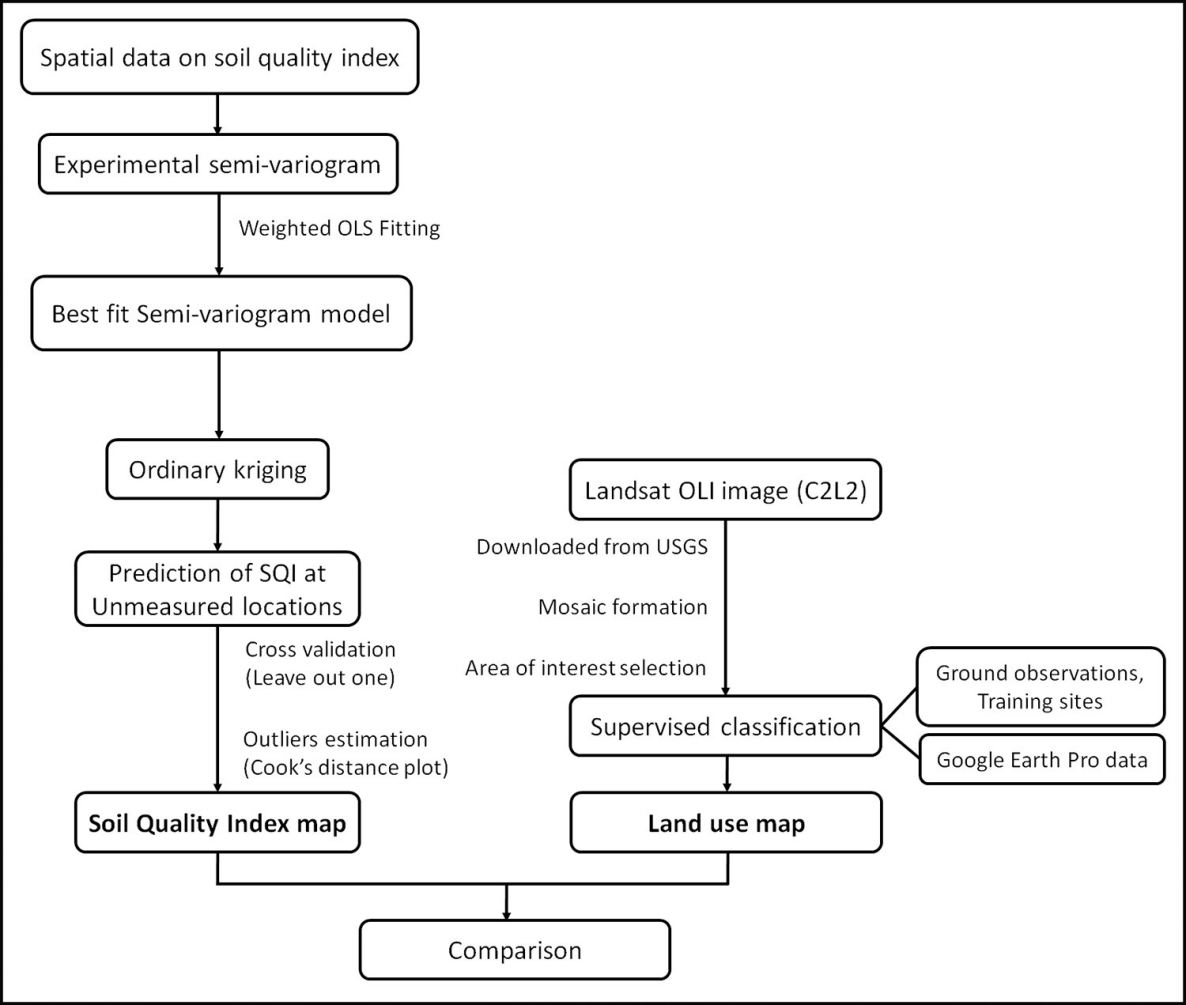
Flow-diagram explaining the process of mapping the soil quality index and land uses over the study area.

Next, the fitted semi-variogram parameters corresponding to best fit model were used to create SQI map of the study area through ordinary kriging (Fig 2). Estimates of SQI values at unsampled locations, z(u), were computed as following [45, 47]:

$$z(u)=\sum_{n(u)}^{a=1}\lambda_{a}z(u_{a})$$ (7)

where, z(u) is the kriging estimated values of SQI at any unsampled site, based on values of known location z(u_a_) located in close neighborhood and *λ*_*a*_ is weight assigned to z(u_a_). To understand the existence of outliers, Cook’s distance plot was calculated. Leave-out one cross-validation was performed between observed and estimated values and correlation coefficient (r), regression coefficient (R^2^) and root mean square error (RMSE) were calculated for that. The SQI spatial variability was compared with the satellite data derived land use map of the area (Fig 2).

## 3. Results

Following the objective, this study adopted a stratified approach to understand the impact of LULC on soil quality. First, different soil indicators were analyzed and a comprehensive SQI was built to quantify the soil quality through a single value. Finally, the spatial distribution of soil quality was observed.

### 3.1. Determination of soil indicators

All the soils were acidic in nature (forest $\bar{x}$: 5.29, converted lands $\bar{x}$: 5.38, croplands $\bar{x}$: 5.59) (Table 1). The dominant number of soils were of sandy loam texture while the presence of loam, silty loam, sandy clay loam and loamy sand were also observed. Soil CEC was found to be low (forest $\bar{x}$: 4.14, converted lands $\bar{x}$: 4.05, croplands $\bar{x}$: 4.19 meq 100 g^-1^ soil) while presence of significant amounts of exchangeable Al was observed. Table 1 indicated no significant impact of land use on soil texture, CEC and exchangeable Al. X-ray diffraction analysis of the bulk soil samples indicated the dominance of muscovite, quartz, zeolite types of minerals in all the soils [26].

**Table 1:** Soil properties under different land uses.

Parameters	Forest		Converted lands		Croplands	
	Mean	Range	Mean	Range	Mean	Range
pH	5.29	4.75–5.73	5.38	4.43–7.83	5.59	4.2–8.04
EC (dS m^-1^)	0.15	0.1–0.2	0.14	0.1–0.2	0.17	0.1–0.3
CEC (meq 100 g^-1^ soil)	4.14	1.54–6.94	4.05	1.56–6.89	4.19	4.09–7.21
Exchangeable Al (meq 100 g^-1^ soil)	0.22	0.11–0.28	0.15	0.10–0.21	0.18	0.11–0.29
Texture						
Clay (%)	14.53	6.0–18	15.76	4.0–26	14.29	4.0–24
Silt (%)	22.05	2.0–42	21.04	2.0–48	22.91	2.0–64
Sand (%)	63.31	43–87	63.20	30–92	63.64	20–90
Soil total C (%)	1.21	0.52–2.87	0.87	0.60–1.37	0.79	0.19–1.05
Soil organic C (g Kg^-1^)	11.61	4.85–28.13	8.25	5.71–13.12	7.30	0.963–14.42
Available N (Kg ha^-1^)	147.3	85.3–198.2	188.4	50.2–323.6	202.7	109.1–281.0
Available P (P_2_O_5_ Kg ha^-1^)	51.7	13.7–260.8	77.9	23.5–182.6	80.3	12.7–559.9
Density fractionation of soil						
Heavy fraction (g kg^-1^)	965.82	954.56–976.69	970.60	962.66–979.88	968.55	957.27–980.59
Light fraction (g kg^-1^)	2.43	0.31–5.35	1.94	0.12–3.34	1.20	0.41–2.73
(Light fraction/ Heavy fraction) × 100	0.25	0.03–0.54	0.20	0.01–0.34	0.12	0.04–0.27
C associated within density fraction						
Heavy fraction (g kg^-1^)	11.4	6.32–13.59	8.41	6.23–11.78	7.88	6.72–11.14
Light fraction (g kg^-1^)	259.53	212.5–767.51	188.33	80.36–388.14	188.03	29.11–319.28
(C in Heavy fraction/Light fraction) × 100	4.15	3.69–4.31	5.78	2.58–17.15	5.37	2.73–28.31
Soil aggregation						
Mean Weight Diameter (mm)	1.40	0.62–1.8	1.35	0.96–1.72	1.31	0.35–1.81
Geometric Mean Diameter (mm)	1.01	0.58–1.23	0.97	0.73–1.19	0.95	0.48–1.23
Macroaggregate associated C (g kg^-1^)	12.11	7.83–16.04	11.05	7.16–17.61	8.55	3.93–15.10
Microaggregate associated C (g kg^-1^)	12.21	4.11–16.62	11.33	6.82–20.81	9.14	3.32–16.05
Microbial biomass C (μg g^-1^)	73.35	4.36–261.08	36.45	5.47–346.97	25.94	1.86–77.99
Microbial quotient	0.72	4.99–2.73	0.44	0.64–3.73	0.45	0.01–4.36
Fluorescein Di-Acetate (μg g^-1^ h^-1^)	710.66	166.02–2207.15	464.71	235.44–898.62	454.47	17.87–1062.70

Total C was found much higher in the forest soils ($\bar{x}$: 1.21%) than the converted lands ($\bar{x}$: 0.87%) and cropland soils ($\bar{x}$: 0.79%). On a similar note, soil organic C was highest in forests ($\bar{x}$: 11.61 g Kg^-1^), followed by soils of converted lands ($\bar{x}$: 8.25 g Kg^-1^) and croplands ($\bar{x}$: 7.30 g Kg^-1^). Cropland soils were high in available N and P ($\bar{x}$: 202.7 Kg N and 80.33 Kg P_2_O_5_ ha^-1^) in comparison to soils of converted lands ($\bar{x}$: 188.4 Kg N and 77.9 Kg P_2_O_5_ ha-1) and forests ($\bar{x}$: 147.3 Kg N and 51.7 Kg P_2_O_5_ ha^-1^).

In density fractionation of soils, 96.9±1% soil mass was recovered. Nearly the entire amount of the soils was found within the heavy density fractions while only small quantity of soils were separated as light density fractions. A ratio of these two fractions ((light fraction/ heavy fraction) × 100) indicated a comparatively higher presence of light fractions in forest soil, followed by soils of converted lands and croplands (Table 1). A very high amount of C was found to be associated with the light density fractions in all the soils while C associated with heavy density fractions was much less. Soil aggregate stability was estimated using MWD and GMD under all the three LULC classes. Both of these indices confirmed a higher presence of soil macroaggregate in forest soils ($\bar{x}$: MWD: 1.40, GMD: 1.01), followed by converted land soils ($\bar{x}$: MWD: 1.35, GMD: 0.97) and soils of croplands ($\bar{x}$: MWD: 1.31, GMD: 0.95). Among the different LULC classes, forest soils were found to hold maximum C within aggregates (macro: $\bar{x}$ 12.11 g kg^-1^; micro: $\bar{x}$ 12.21 g kg^-1^). Lowest aggregate occluded soil C was observed under croplands (macro: $\bar{x}$ 8.55 g kg^-1^; micro: $\bar{x}$ 9.14 g kg^-1^) (Table 1).

Forest soils also represented a much higher microbial biomass C ($\bar{x}$ 73.35 μg g^-1^) and microbial quotient ($\bar{x}$ 0.72) in comparison to the soils of converted lands (microbial biomass C: $\bar{x}$ 36.45 μg g^-1^, microbial quotient: $\bar{x}$ 0.44) and croplands (microbial biomass C: $\bar{x}$ 25.94 μg g^-1^, microbial quotient: $\bar{x}$ 0.45) (Table 1). Soil enzymatic activities (as represented by FDA-HR) were also found low in the soils of croplands ($\bar{x}$ 454.47 μg g h-1) and converted lands ($\bar{x}$ 464.71) in comparison to forests ($\bar{x}$ 710.66).

### 3.2. Soil indexing and mapping

**Fig 3:**
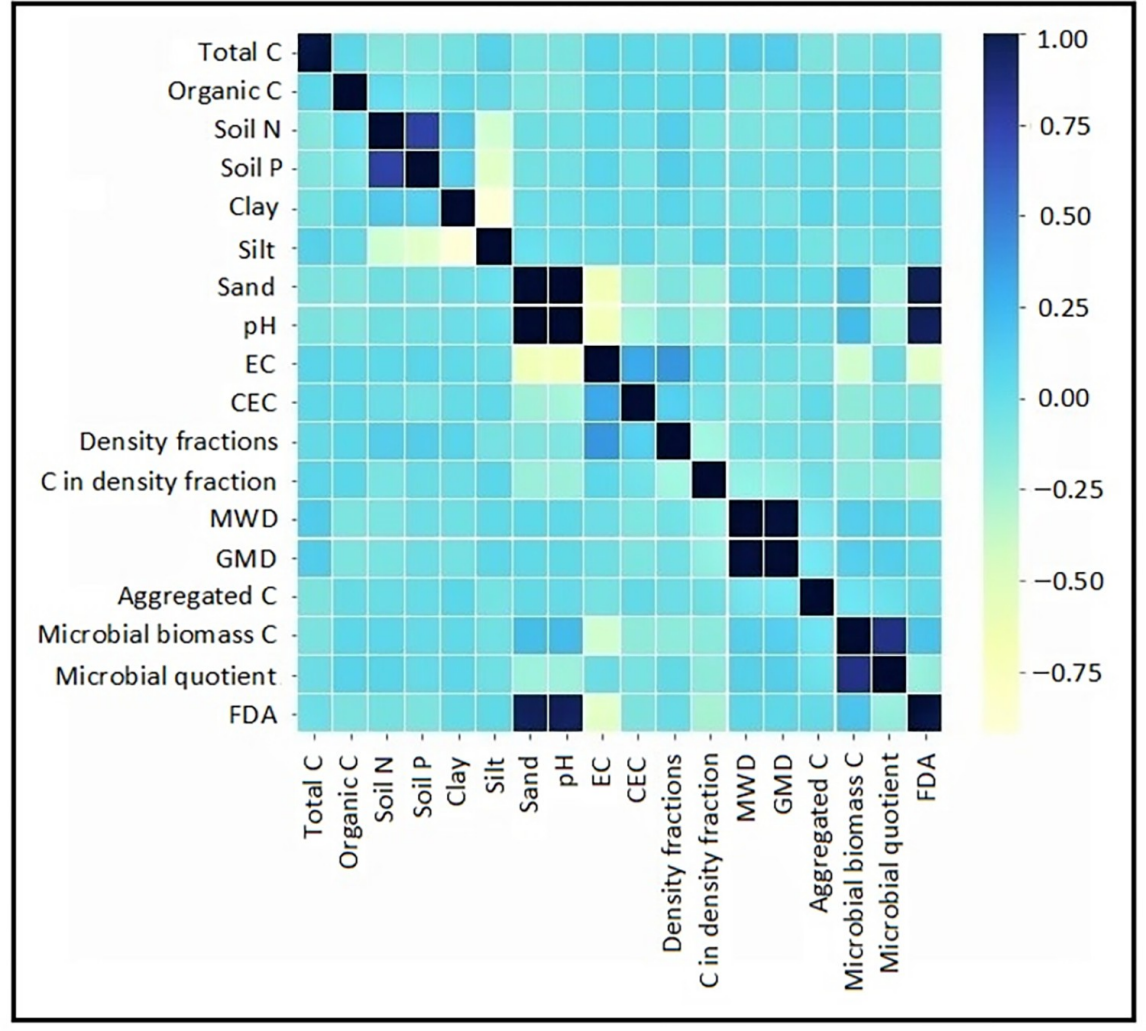
Heatmap of variables showing Pearson’s correlation coefficient.

**Fig 4:**
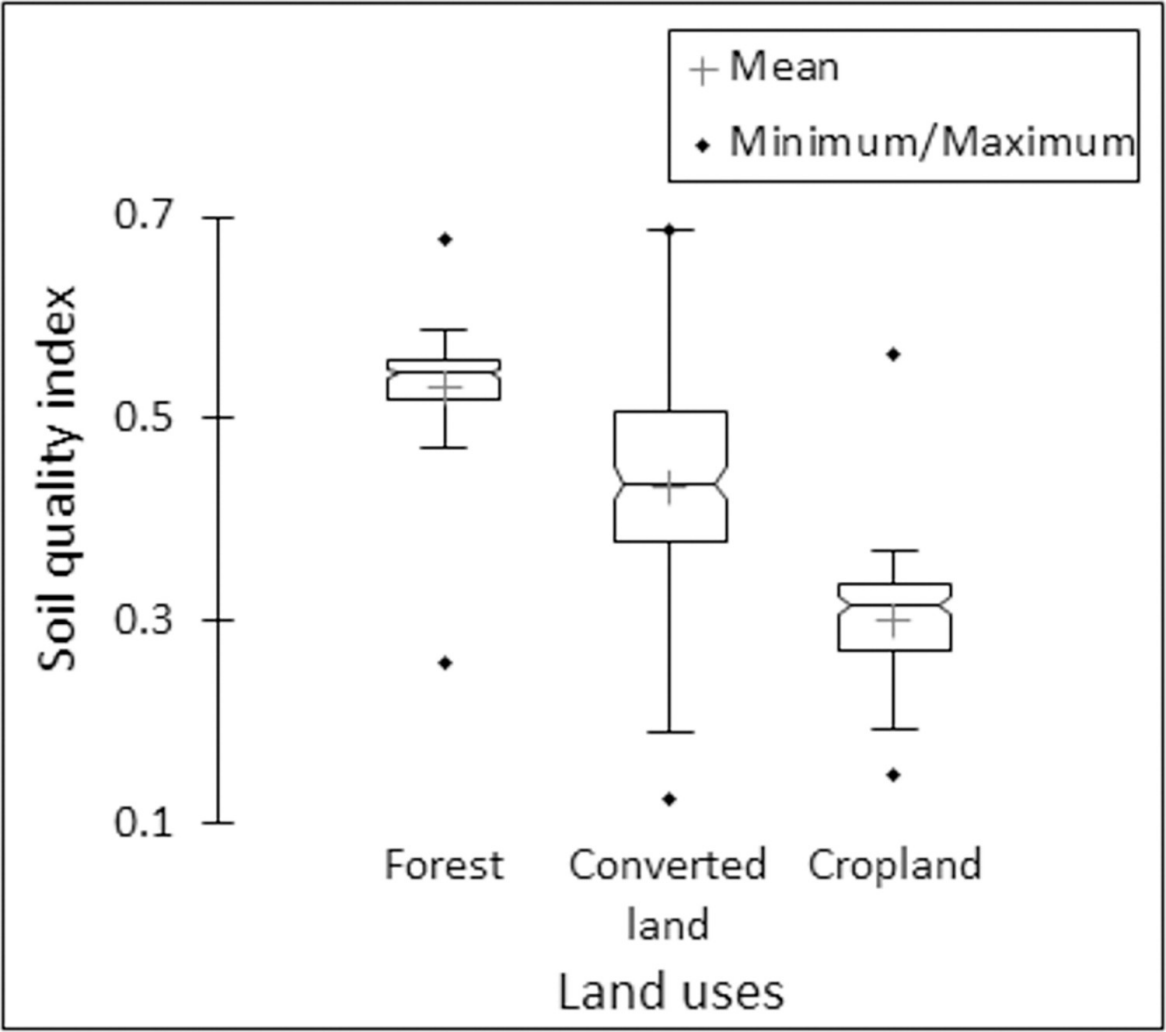
Soil quality index scores under different land use classes.

To create soil quality index, the principle of MDS was followed in this study. A total of 19 indicators (Table 2) were considered for SQI formation. It was further reduced to 8 indicators following high eigenvalues (≥ 1) obtained in PCA. Selection of soil indicators for MDS under these 8 PCs was made using eigenvectors (Table 2) and the sorted soil indicators were (i) density fractionation of soil, (ii) FDA, (iii) microbial biomass C, (iv) available P, (v) C associated within density fraction, (vi) available N, (vii) ratio of macroaggregated and microaggregated C and (viii) total C. Among these, available N (eigenvector 0.843) and available P (eigenvector 0.778) were showed high correlation (Pearson’s correlation coefficient >0.6) (Fig 3) and only available N was kept in MDS following its higher eigenvector loading (Table 2) [42, 43]. Table 3 showed the relative proportion of these indicators for the calculation of a weighted additive index. The SQI score-sheet indicated that forest soils ($\bar{x}$ 0.532) were best in quality, followed by soils of converted lands ($\bar{x}$ 0.432) and croplands ($\bar{x}$ 0.301) (Fig 4). However, soils of converted lands exhibited a more stretched range of SQI (0.124 to 0.687) in comparison to forest soils (0.257 to 0.678) and soils of croplands (0.147 to 0.563).

**Table 2:** Selection of parameters (indicators) for soil quality index using eigenvectors obtained through principal component analysis.

Parameters	PC1	PC2	PC3	PC4	PC5	PC6	PC7	PC8
pH	-0.001	0.012	-0.001	0.032	0.049	0.054	-0.123	0.166
EC	0.023	-0.055	-0.145	0.008	0.003	0.005	0.000	0.004
CEC	0.011	-0.016	0.484	0.005	0.002	-0.001	-0.001	0.001
Exchangeable Al	0.015	-0.001	0.041	0.002	0.007	0.000	0.002	0.008
Clay	0.001	0.000	0.007	-0.500	0.071	-0.212	0.003	0.063
Silt	-0.001	0.001	-0.010	-0.319	0.067	-0.200	0.008	0.060
Sand	0.000	0.001	0.000	0.003	0.005	0.005	-0.010	0.016
Soil total C	0.000	0.000	-0.001	-0.013	0.011	-0.017	-0.603	***0*.*762***
Soil organic C	0.000	0.000	0.000	-0.001	0.002	0.002	0.000	0.003
Available N	0.000	0.000	0.002	0.201	-0.022	***0*.*843***	-0.028	0.046
Available P	0.001	-0.001	0.000	***0*.*778***	0.098	-0.437	0.009	0.059
Density fractionation of soil ((Weight of Light fraction/ Heavy fraction) × 100)	***1*.*000***	0.008	0.008	-0.001	0.001	0.000	0.000	0.000
C associated within density fraction ((C in Heavy fraction/Light fraction) × 100)	-0.001	-0.002	-0.007	-0.017	***0*.*988***	0.088	0.026	-0.017
Soil aggregate—Mean Weight Diameter	0.000	0.000	0.000	0.000	-0.018	-0.019	-0.031	0.028
Soil aggregate—Geometric Mean Diameter	0.000	0.000	0.000	0.000	-0.011	-0.011	-0.016	0.015
Macroaggregated C/ Microaggregated C	0.000	0.000	0.000	-0.001	-0.020	0.030	***0*.*786***	0.613
Microbial biomass C	-0.012	0.023	***0*.*863***	-0.002	0.008	0.000	0.000	0.001
Microbial quotient	0.000	0.000	0.009	-0.005	-0.024	-0.016	0.008	0.010
Fluorescein di-acetate	-0.007	***0*.*998***	-0.020	0.001	0.001	0.000	0.001	-0.002

PC: Principal component

(Bold texted cells indicated selected parameters in PCA and their corresponding eigenvectors)

**Table 3:** Proportion and eigenvalues of the selected indicators for calculation of weighted additive soil quality index.

Indicator parameter	PC*	Variance	Proportion	Cumulative proportion
Density fractionation	1	12844.83	0.572	0.572
Fluorescein di-acetate	2	3660.33	0.163	0.735
Microbial biomass C	3	2604.90	0.116	0.851
Available P **†**	4	1055.43	0.047	0.974
C associated within density fraction	5	943.15	0.042	0.893
Available N	6	763.50	0.034	0.927
Macroaggregated C/Microaggregated C	7	538.94	0.024	0.998
Total C	8	44.91	0.002	1

* Principal component (This proportion of selected parameters were used to calculate weighted additive index); **†** Available P was not considered in final dataset due to high correlation (Pearson’s correlation coefficient >0.6) with available N but low eigenvector loading than available N

**Fig 5:**
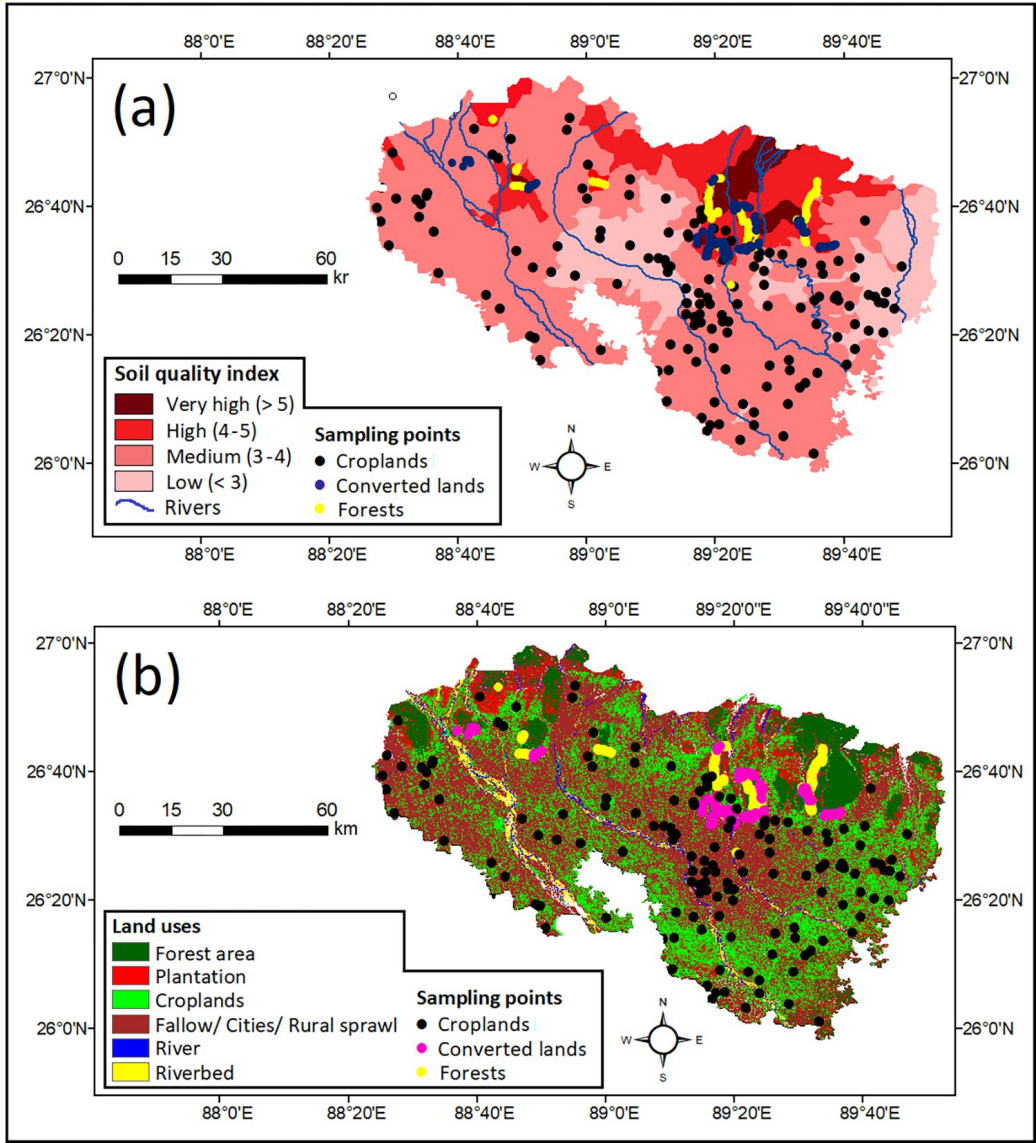
Comparative maps of soil quality index and land uses of the study area.

Ordinary kriging interpolation method was used in this research for spatial mapping i.e. to calculate the values of SQI in unsampled locations [45, 48]. The resultant map indicated a diverse distribution of the soil quality. For easy understanding, the range of SQI has been divided into four classes *viz*. very high (score >5), high (4–5), medium (3–4) and low (<3). The Fig 5A showed that soils collected from forests and converted lands fit into very high or high SQI while medium to low SQI scores were observed under the croplands.

## 4. Discussion

Soils of this area are mainly sandy as all the rivers leave their upper reach here and thus deposit coarse particles. Presence of such soil and very high rainfall (> 3000 mm per year, as per FAO ClimWat database) resulted in leaching of basic cations and acidic soils here. The low pH also possibly consequence noteworthy presence of exchangeable Al [49, 50].

### 4.1. Soil indicators under different LULC

The higher C status in forest soils in comparison to converted and cropland soils was expected as tillage operations cause fast depletion of soil C through mineralization [51–53]. Continuous C deposition in forest soils through leaf and litter fall was another reason for high soil C status. Converted lands, which were mainly distributed along forest fringes, showed lesser amount of C than forest soils but higher C than cropland soils (Table 1). Relatively recent human intervention and less intense cropping practices in converted lands were the possible reasons for this. The higher presence of available N and P in cropland soils can be attributed to the regular application of nitrogenous and phosphatic fertilizers there, as indicated by the field survey.

Physical fractionation of soils was done to comprehend soil structure and C distribution within aggregates and in attachment with mineral matrix [35, 36, 38, 54]. In density fractionation, heavy density fraction represented humified/ amorphous organic matter attached to soil mineral matrix [55] whereas light density fraction represented loose and undecomposed plant/ organic residues [55, 56]. Continuous fresh organic matter addition in forest soils by leaf-litter fall possibly caused the comparative higher light density fractions there. Due to presence of high C in this fresh organic matter, the light fraction carries high amounts of C [57]. The heavy fraction contains a lot less C as only a very small portion ultimately gets humified and sequestrated in soils [58].

There is an accepted scientific perception that forest soils have good aggregation due to its undisturbed ecology [13, 59]. The presence of high organic C in these soils also favors the process since C (or organic matter) is the key to bind soil particles into aggregates [60]. While humic substances and soil particles combine to form microaggregates, microbial polysaccharides, organic mucilages bind the microaggregates into larger macroaggregates [61]. Conversely, tillage operations break-down the macroaggregates in croplands, exposing physically protected C to soil microorganisms and causing C loss from soils [36, 62]. In this study, highest and lowest soil macroagegate formation was observed in forest and cropland soils. As evident from Table 1, clay content did not vary enough to influence this difference in soil aggregation.

The change in LULC also affected soil macro and microaggregate occluded C. Highest aggregate occluded C was found in forest soils followed by soils of converted and croplands. In soils, aggregates protect the encrusted C from microbial decomposition [36, 63]. Therefore, higher soil aggregation leads to a longer turnover time of C or pathway of its sequestration [61]. This study, therefore, inferred a possible C accrual process in the forest soils.

In this research, microbial activities were represented by FDA-HR as different microbial enzymes (like lipase, esterases, protease etc.), responsible for soil organic matter decomposition, get involved in it FDA-HR [40, 64]. The high microbial biomass C and microbial activity in forest soils can be explained by easy availability of soil organic C, the food and energy source of soil microbes [29]. In addition, healthy ecology, permanent soil cover and no anthropological perturbation in forest soils might also have favored better microbial proliferation [65]. Other studies also found low microbial biomass C in agricultural soils or in soils of converted lands (from native forests to croplands or tea plantations) due to disturbances and degradation of overall soil health [66–68].

### 4.2. Evaluation of soil quality through indexing

Evaluation of overall soil quality is important for identifying soil’s potential to perform under different land uses [7]. Estimation of soil quality under different co-existing LULC is also important as uncontrolled land use change and farming results in degradation of soil quality [22, 69, 70]. In this study, MDS was used to synthesize SQI, which can lead to a single unique quantitative value as a decision tool for soil quality [45]. Only 7 indicators were selected through PCA technique after correlation test, as detailed earlier. The finally prepared additive index has the potential to avoid the complexity of expressing different indicators in separate numerical scales through data normalization [7, 42].

The lowest SQI score of cropland soils (Fig 4) was possible due to faulty management practices (like high cropping intensity, excessive tillage, absence of legumes in crop rotations, cultivation of heavy feeder crops etc.) [24, 26]. The comparatively better soil quality of the converted lands (than croplands) at forest fringe was possibly due to less intensive cropping practices (in lands converted from forest to croplands) or sustainable, less exhaustive soil use (in lands converted from forest to new tea plantations) and enduring inherited forest soil health [71]. The diverse duration and type of the conversion process possibly caused a wide range of soil quality in converted lands. Following the objective, this study confirmed the best soil quality under forest cover and a process of soil degradation under continuous cultivation practices. The constant and sharp decline of SQI in converted lands and croplands indicated that sustainable management practices were not followed. This might restrict the soils to perform their maximum and diverse ecosystem services in the future [7, 72].

### 4.3. Spatial distribution of soil quality

Understanding the spatial distribution of soil quality is important as heterogeneity of LULC and diversity of management practices can influence soil properties a lot [4, 10, 19]. The SQI map, prepared by ordinary kriging interpolation, indicated a better soil quality in forests followed by soils of converted and croplands (Fig 5A). As results indicated a clear connection of soil quality with LULC of the area, a comparison was drawn between the two (Fig 5A and 5B). The land use was found to be distributed into six distinct classes *viz*. forest area, plantation (mainly tea gardens), croplands, fallow/ cities/ rural sprawls, rivers and riverbeds (Fig 5B). Among these, croplands and agricultural fallows swap their roles seasonally while rivers and riverbeds do not contain soils. This comparison confirmed that soils of croplands, fallows, urban and rural areas had medium to low SQI while areas entirely under forest showed high to very high SQI scores. Soils of tea gardens and croplands near the forest fringe (i.e. converted lands) showed medium to high SQI in most places. This study portrayed the spatial distribution of soil quality in association with the LULC of the area. A clear declining trend of soil quality was observed as forest> converted lands> croplands.

This study represents a typical example of soil quality degradation due to land use change and faulty management practices. An increase in population pressure, need for more agricultural lands for food security, un-planned new tea plantations have wiped out a large portion of the natural forests of this area since the last century [23, 24, 73]. Although governmental and non-governmental protection measures have cut down the deforestation rate manifold, the practice is still going on [23]. These changes of LULC have affected the soil quality of this region, as indicated in this study. Besides, the cultivation of heavy feeder crops (like maize), application of straight fertilizers, high cropping intensity, heavy tillage etc. have caused an unstable soil quality in the croplands [74, 75].

## 5. Conclusions

The scenario presented in this paper is not an exclusive event. While anthropogenic pressure is changing land uses and soil quality everywhere in India, faulty management practices degrade croplands’ soil quality. This is a matter of great concern as Indian soils are inherently poor in health and hold very low soil C due to tropical sub-tropical climate and huge soil erosion. Proper soil management and preservation of soil quality is a serious issue here to ascertain future food security. Indian national parks and sanctuaries are already protected by the laws. However, to protect open forests and trees outside forestry and to restrict further soil degradation through land use change, holistic initiatives from governmental, international, quasi-governmental, private-public sectors are required. Community management (involving local indigenous people) can also be an approach for land use and soil quality conservation in forest fringes. Sustainable management practices in croplands like more organic manure dependency, minimum tillage also should be assured along with regular monitoring to uplift the cropland soil quality.
